# Dr. Ultzman’s Treatment of Functional Impotence

**Published:** 1885-09

**Authors:** 


### Selections.

### Dr. Ultzman's Treatment of Functional Impotence.

The two forms of this affection which present themselves most frequently for treatment are: 1st, Psychical impotence, and, 2d, Impotence from two early ejaculation of the seminal fluid.

They occur usually in strong, healthy, young men, and are the forms which yield readily to proper treatment.

The treatment is almost entirely local, as the difficulty consists in the incapability of having a normal erection. The capacity of exciting erections resides in the prostate; hence, this is the point to which treatment is to be directed. This is of several forms, all calculated to relieve the hyperæsthesia of the prostatic urethra, and excite the prostate to the production of powerful erections.

The simplest form of treatment is by passing a steel sound into the bladder daily, leaving it there from five to ten minutes, at the same time depressing the handle so as to make pressure upon the prostate. Usually, in a few days or weeks, powerful erections are excited. The use of astringents upon the prostatic urethra is another method. The remedies used are either tannic acid or solution of nitrate of silver. Tannic acid is inserted in small slender suppositories by the *porte remède* and deposited in the prostatic portion. Urine should not be passed for half an hour after the insertion. This treatment may be used every day, and not less frequently than every second day, till normal erections occur. Nitrate of silver is used in a five per cent. solution, and three or four drops deposited upon the prostatic portion by a deep urethral syringe, once every three or four days. During treatment, the patient should abstain from all sexual excitement.

